# A RhoC Biosensor Reveals Differences in the Activation Kinetics of RhoA and RhoC in Migrating Cells

**DOI:** 10.1371/journal.pone.0079877

**Published:** 2013-11-05

**Authors:** Jon S. Zawistowski, Mohsen Sabouri-Ghomi, Gaudenz Danuser, Klaus M. Hahn, Louis Hodgson

**Affiliations:** 1 Department of Pharmacology and Lineberger Cancer Center, University of North Carolina School of Medicine, Chapel Hill, North Carolina, United States of America; 2 Department of Cell Biology, The Scripps Research Institute, La Jolla, California, United States of America; Karolinska Institutet, Sweden

## Abstract

RhoA and RhoC GTPases share 92% amino acid sequence identity, yet play different roles in regulating cell motility and morphology. To understand these differences, we developed and validated a biosensor of RhoC activation (RhoC FLARE). This was used together with a RhoA biosensor to compare the spatio-temporal dynamics of RhoA and RhoC activity during cell protrusion/retraction and macropinocytosis. Both GTPases were activated similarly at the cell edge, but in regions more distal from the edge RhoC showed higher activation during protrusion. The two isoforms differed markedly in the kinetics of activation. RhoC was activated concomitantly with RhoA at the cell edge, but distally, RhoC activation preceded RhoA activation, occurring before edge protrusion. During macropinocytosis, differences were observed during vesicle closure and in the area surrounding vesicle formation.

## Introduction

The Rho GTPase phylogenetic subfamily in mammals is comprised of RhoA, RhoB and RhoC, which share 85% overall amino acid identity. Northern blotting indicates that all are ubiquitously expressed, though expression levels vary greatly [1]. Although RhoA and RhoC share 92% identity, they have markedly different roles in motility and cancer. RhoA regulates actin polymerization, Rac activity, and actomyosin contractility [2–4] while RhoC has primarily been linked to formin-mediated protrusion, invadopodia and cancer cell invasion[4–7]. RhoA and RhoC have reciprocal roles in controlling cancer cell motility. RhoC knockdown has been effective in suppressing metastasis in xenograft models [8], while knockdown of RhoA leads to enhanced invasion [5]. In cell culture models, activators of RhoC induce loss of cell polarity and increase invasion, while activation of RhoA inhibits invasiveness as well as motility [5]. To better understand these differential functions of RhoA and RhoC we developed a biosensor for RhoC, and used it together with an established RhoA biosensor [9,10] to elucidate the different spatio-temporal dynamics of RhoA and RhoC during protrusion and macropinocytosis. 

## Materials and Methods

### Biosensors

RhoC FLARE was created by linking ROCK1 residues 905-1046 to monomeric Cerulean [11], an unstructured linker of optimized length [12], monomeric Venus [13], and full-length RhoC (Figure S1; Appendix S1). The construct was subcloned into pTriEX-HisMyc4 (Novagen) for transient expression. For linker optimization, repeating units of TSGSGKPGSGEGSTKGGS were cloned between the two fluorescent proteins and tested for optimal FRET/CFP ratio change. We found that a biosensor with 4 linkers produced the largest dynamic range. Characterization of biosensor responses was carried out as described previously [9]. Briefly, HEK293T cells were plated overnight at 1.25x10^6^ cells/well of 6-well plates coated with poly-L-lysine, and transfected using Lipofectamine2000 reagent (Invitrogen) following the manufacturer’s protocols. The biosensor and the regulator cDNAs were co-transfected at ratios of 1:4 for the biosensor and the GDI or the GAP and 1:4:1 - 10 for the biosensor:GDI:GEF. Forty eight hours following the transfection, cells were trypsinized and suspended in ice cold PBS, and then placed directly into fluorometric cuvettes to measure fluorescence emission spectra. The spectra were obtained by exciting cold, live, 293 cell suspensions in the cuvette with 433nm light, with emission scanned from 450 - 600nm. The fluorescence reading of a sample cell suspension with empty cDNA (pCDNA3.1) was used to measure light scatter and autofluorescence, which were subtracted from the data. The resulting spectra were normalized to the peak CFP emission intensity to generate the final ratiometric spectra.

### Cell culture

MEF/3T3 (Clontech) were maintained in Dulbecco’s modified Eagle’s medium (Gibco) with 10% FBS. To induce RhoA biosensor expression, 2µg/ml doxycycline was removed 48 hours prior to imaging by detaching cells through brief trypsinization and then replating them at 10^4^ cells per 10cm dish. A stable cell line expressing RhoC was produced using a tet-inducible retroviral system as previously described [9]. Cells were plated on fibronectin-coated glass coverslips (10 μg/ml) for 3 hours prior to imaging. Imaging was performed in Ham’s F-12K without phenol red (Biosource), 10 mM HEPES and with 2% FBS in a heated closed chamber. For serum-stimulation experiments, cells were starved for 24hrs in medium containing 0.5% serum, and stimulated with medium containing 10% serum.

### Imaging

Activation levels of RhoA and RhoC were measured by monitoring the ratio of ECFP or mCerulean emission to FRET emission. Images were acquired using a custom microscope capable of simultaneous acquisitions of FRET with either ECFP or mCerulean, through two CoolsnapES2 cameras mounted via a beamsplitter. The specifications of this imaging system are detailed elsewhere [14]. Images acquired by this two camera system were properly aligned using *a priori* calibration and morphing to achieve accurate pixel-by-pixel matching as described previously [15]. Image processing, ratio calculations and correction for photobleaching were as described previously [9]. 

### Morphodynamic correlation and computational multiplexing analysis

To analyze the spatiotemporal correlation of RhoC and RhoA activity with cell edge motion, activities were sampled in reporter windows of 2.5 µm width and 0.9 µm depth, maintained at a constant distance from the cell edge. Windows were sampled at different distances from the edge, in steps of 0.9 µm. In addition, the velocity of the edge was determined, enabling the correlation of signaling and morphodynamic activity in a cell-frame of reference amenable to statistical comparisons, as previously described [16]. Temporal cross-correlation between RhoC or RhoA activity and edge velocity was determined for all reporter windows, examining correlation at time differences up to a maximum of 500 seconds (50 frames). Characteristic cross-correlations between RhoA or RhoC activation and edge velocity were estimated by fitting a smoothing spline to the combined pool of cross-correlations. The variance and 95% confidence interval of the smoothing spline approximation, and hence of the location of the maximum correlation, was calculated using a non-parametric bootstrap method [17]. Because of the invariance of the reporter window shape, sampling data from multiple cells could be pooled in this analysis. The “timelag” as calculated and presented is the highest peak of the cross-correlation trace at a given distance from the edge. The region within 0 - 3 pixels from the edge usually contains significant errors due to fluorescence image threshold masking from the associated lower signal to noise ratio at the edge. Thus we considered the region 3 - 6 pixels from the edge as the beginning of reliable edge measurements in our correlation analysis.

## Results and Discussion

**Figure 1:**
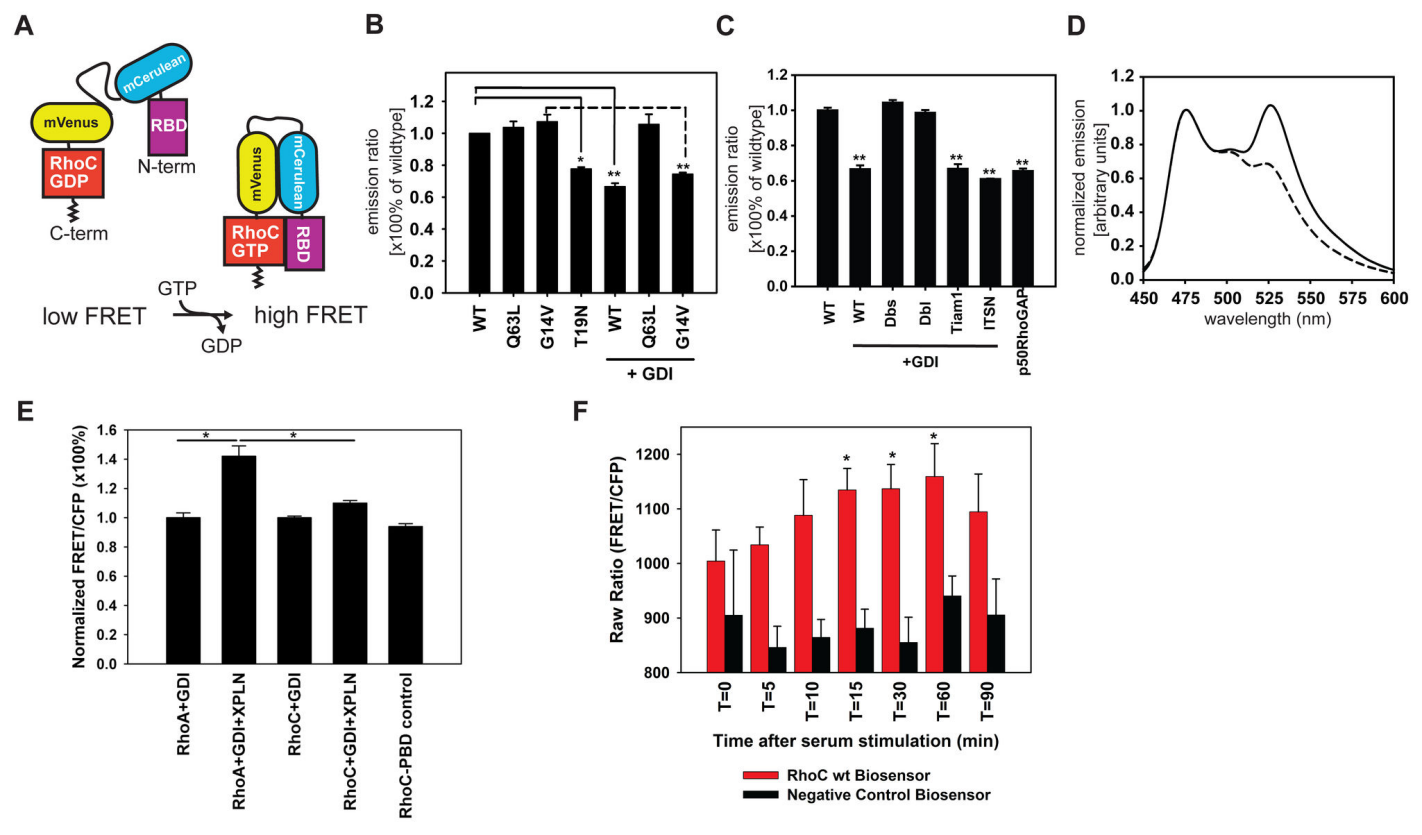
Construction and validation of the RhoC FLARE biosensor. (**A**) Design of the biosensor (**B**) Analysis of cell suspensions expressing biosensor mutants. RhoC G14V but not Q63L is susceptible to Rho GDI. Data shown with S.E.M. n=3. (**C**) RhoC biosensor cell suspensions co-expressing Rho GEFs (Dbs, Dbl), Rac-specific GEF Tiam1 and Cdc42-specific GEF intersectin. Results were normalized to wildtype=1, mean of at least three independent experiments. Error bars represent standard error of the mean, ** = *P* < 0.001 and * = *P* < 0.05 (t-test, one-tailed), for **B** and for **C**. (**D**) 293T cell suspensions expressing RhoC biosensor with (dashed curve) or without (solid curve) RhoGDI coexpression (excitation at 433 nm). (**E**) RhoA and RhoC biosensor cell suspensions co-expressing Rho GDI and the RhoA/B-specific GEF XPLN. The normalized emission ratio of a negative control biosensor harboring p21 binding domain (PBD) of p21 activated kinase 1 (PAK1) instead of the Rho binding domain (RBD) from ROCK is shown in the absence of GDI overexpression. * = p < 0.001 (t-test, one-tailed), n=3, data shown with SEM.(F) Raw emission ratios of cells stably expressing wildtype RhoC FLARE.sc or control RhoC-PBD biosensors following serum stimulation for the indicated timepoints. * = p < 0.05 compared to t=0 (t-test, one-tailed), n=10 at each time point, data shown with SEM.

The design of the new RhoC biosensor was similar to that of our previously published biosensor for RhoA [9], but incorporating RhoC , a different set of linkers, and a binding domain from ROCK1 (RBD, amino acids 905-1046) that preferentially binds to GTP-loaded RhoC [18]. The domain, at the amino terminus of the biosensor, was fused to monomeric Cerulean fluorescent protein [11], followed by an optimized linker, monomeric Venus [13], and finally full-length RhoC. Upon GTP-loading, the RBD bound to the RhoC, increasing FRET (Figure 1A). The two fluorophores were placed on the internal portion of the biosensor chain, leaving the C-terminus of the GTPase intact for binding and regulation by Rho guanine nucleotide dissociation inhibitor (RhoGDI). Consistent with recent nomenclature we have introduced to differentiate biosensor designs, the new biosensor is named RhoC FLARE.sc (sc denotes a single chain design) [19,20].

The size of the biosensor precluded purification for *in vitro* characterization, so it was expressed and analyzed in suspensions of HEK cells (Figure 1B-E). The biosensor showed a 41% increase in FRET ratio between inactive and constitutively active mutants (T19N versus Q63L and G14V respectively), with wt biosensor subject to regulation in the cells and therefore responding between these two extremes. The biosensor was expressed in HEK293T cells (see M&M) at high levels to produce sufficient signal for fluorometry of cell suspensions. At these expression levels, cellular negative regulators including endogenous GDI were overwhelmed [9,21], as endogenous ratios of Rho GTPases to GDI in most cells are 1:1 or at most 1:1.1 [22]. Excess wildtype biosensor was observed to translocate to the plasma membrane, where it could encounter active GEFs and become activated. This resulted in the observed high levels of FRET [9]. This effect was reversed by expressing GDI together with the biosensor. The GDI cDNA concentration was titrated (data not shown) to the lowest possible GDI expression levels that could produce maximal suppression of biosensor activity (1:4 ratio of biosensor:GDI cDNA during transfection). This treatment affected the biosensor that had an activating mutation known to be sensitive to GDI (G14V), but not the Q63L mutant that does not bind GDI [22]. Figure 1C shows that GDI effects could be counteracted by expressing excess guanine nucleotide exchange factors (GEFs), but only when using GEFs or GEF fragments that were specific for Rho (DH-PH, activated GEF fragments from Dbs and Dbl, but not from Tiam1 and ITSN [23]). In these fluorometric validation measurements, we overexpressed either full-length or truncated, constitutively active GEF fragments in HEK293T cells. At the high expression levels used it was likely that GEFs were not normally localized, overwhelming native binding sites. In contrast, during later imaging of adherent cells, when expression was kept at the lowest levels providing acceptable signal/noise, endogenous GEFs activated the biosensors. This led to localized GEF interactions, which has been previously shown to impart selectivity for activating RhoA versus RhoC [7,24]. In the cell suspension assays, the fluorescence ratio of the wild type biosensor was also reduced by p50RhoGAP in the absence of excess GDI. Together these data demonstrate robust response of the biosensor to RhoC activation, and specific response to the three major classes of regulatory proteins: GEFs, GAPs and GDI. Unlike Rho family biosensors that are anchored permanently to the plasma membrane, the intact GTPase C terminus in RhoC FLARE as well as in the previous RhoA biosensor allows for interaction with GDI. This is important because it enables our biosensor to reflect control of activation through translocation between the cytoplasm and the plasma membrane [9], which requires interaction with GDI. The FLARE biosensors therefore report GTPase activation rather than the balance of GEF/GAP activity at the plasma membrane. 

We examined whether XPLN, the GEF previously reported to activate RhoA but not RhoC [25], could differentiate between our RhoA [9] and RhoC FLARE biosensors. RhoA activity was rescued upon overexpression of full length XPLN in cells expressing RhoA sensor together with GDI (at a cDNA transfection ratio 1:4:4 for biosensor:GDI:XPLN) (Figure 1E). The same overexpression condition had only modest effects on the RhoC biosensor response. We tested for the specificity of the binding domain used in the RhoC biosensor by changing the Rho kinase binding domain to the p21 binding domain (PBD) from p21 activated kinase 1 (PAK1), a downstream target of Rac1 and Cdc42 [26]. Even in the absence of exogenous GDI, this control biosensor showed a FRET ratio similar to that produced when the normal biosensor was exposed to excess GDI (Figure 1E). 

**Figure 2:**
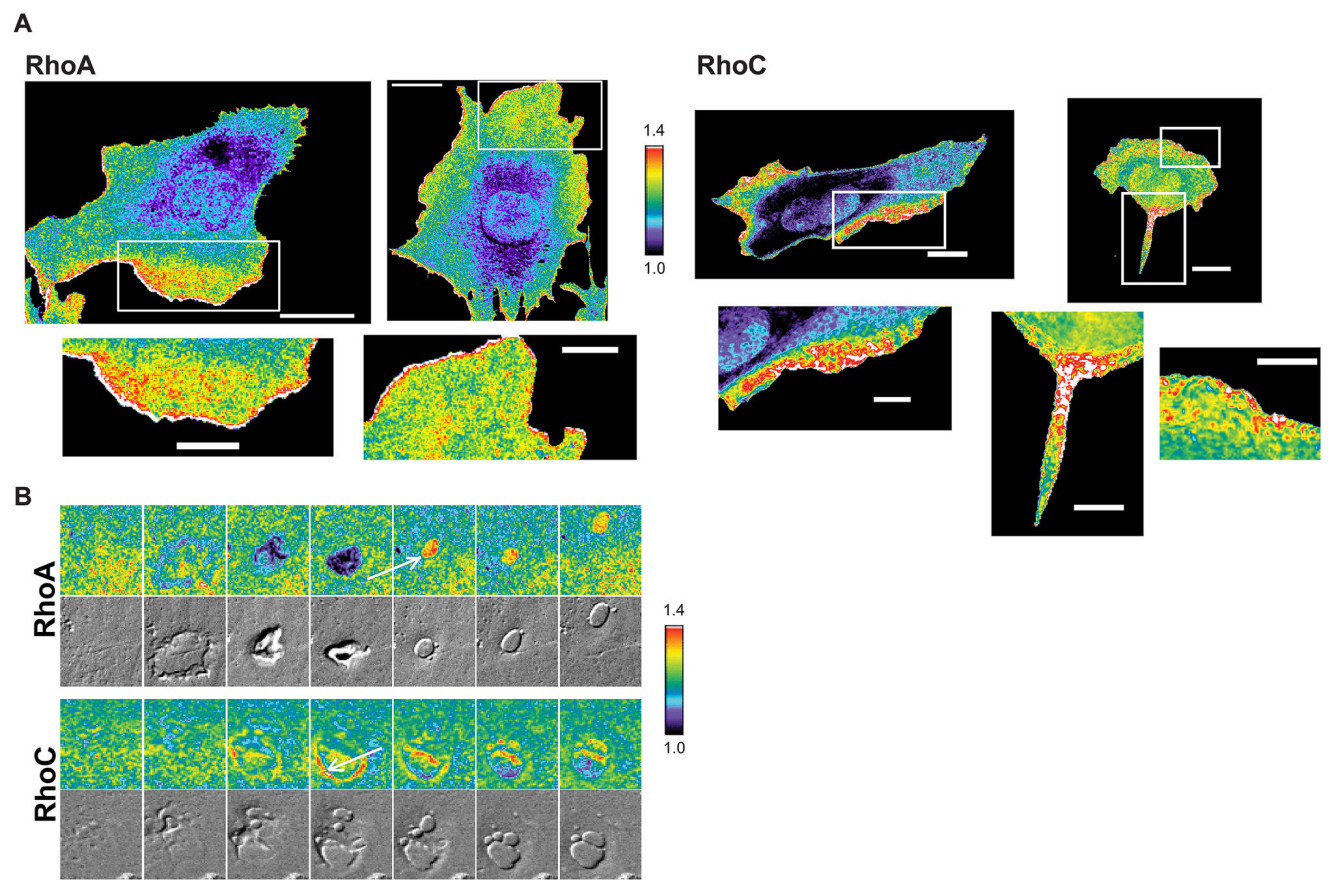
Imaging of the RhoC FLARE.sc biosensor (A) RhoA (left) and RhoC activity (right) in MEFs plated on fibronectin. RhoA scale bar = 30 µm, 15 μm in zoomed images RhoC scale bar = 20 µm, 10 μm in zoomed images. Note the fine band of white/red at the edge of RhoA cells. (**B**) Biosensor emission ratios and corresponding DIC images during macropinocytosis. Arrows highlight RhoA activity during vesicle closure and RhoC activity in the actin cup prior to closure. Width of each panel is 20 µm.

We examined response of the RhoC biosensor to exogenous stimulation [27], using mouse embryonic fibroblasts (MEFs) stably expressing either the wildtype RhoC biosensor or the PBD-control version of the biosensor under an inducible promoter. Upon serum stimulation following 24hr starvation, we saw robust activation of RhoC with the wildtype RhoC biosensor (Figure 1F) but not with the PBD control. The MEFs also showed clear differences in the localization of RhoA and RhoC activity. The RhoA biosensor was maximally activated in a narrow band 0-1 microns from the edge of extending protrusions (Figure 2A) while RhoC showed more heterogeneous and diffuse activation throughout the region from the edge to several microns away from the edge (Figure 2A; Movies 1-4). 

**Figure 3:**
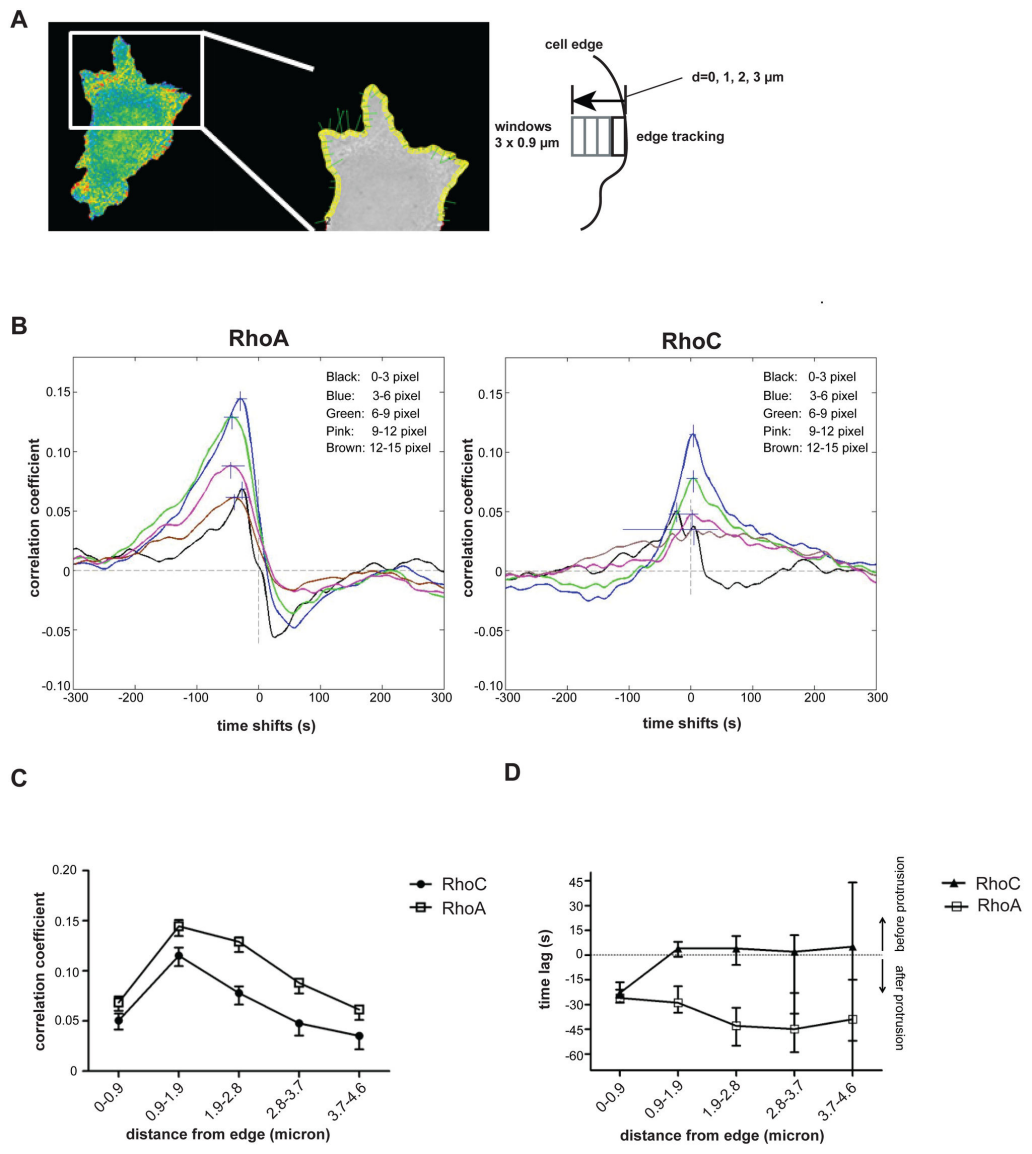
Correlation of RhoA/C activation and cell edge dynamics. (**A**) Top: 2.5 µm width, 0.9 µm depth sampling windows (yellow) are placed at fixed distances from the edge of a MEF expressing the RhoC biosensor. (**B**) Correlation of each biosensor and cell edge velocity as a function of time and distance from the edge. Inset legend indicates color code for spatial zones. RhoA: n=16 cells, 993 windows, RhoC: n=16 cells, 869 windows. Correlation coefficients (**C**) and time shifts (**D**) for RhoA (open) and RhoC (solid) at different distances from the edge. Error bars represent 95% confidence intervals, estimated by bootstrap analysis of variation in the correlation functions.

There were also striking differences between RhoC and RhoA during macropinocytosis (Figure 2B). As reported previously, RhoA activation was attenuated in the actin-rich ring structure that precedes vesicle closure, and a burst of RhoA activity appeared on the vesicle after closure [9]. In contrast, RhoC activity was high in the ring prior to closure, and no burst of activity was observed (Movies S5 and S6; n=5 events). With T19N dominant negative control biosensors, RhoC showed no activation in the ring (n= 5 events) and the burst of RhoA activation was reduced by 50% (WT: average 29.35% change, n=14 events, SD 7.18; T19N: average 15.66% change, n=12 events, SD 6.74).

We focused on comparing RhoC and RhoA activation in the constitutive protrusions of migrating MEFs, where RhoA activity had previously been extensively characterized using the RhoA FLARE.sc biosensor [9,10]. To quantify differences in RhoA and RhoC activity, we turned to the computational multiplexing approach described in Machacek et al., 2009. This method uses cell edge velocity as a common reference to relate, in time and space, the activities of two different biosensors studied in separate experiments. The first step in computational multiplexing is to determine the spatiotemporal correlation between cell edge movement and each of the biosensors separately. As illustrated in Figure 3A and Movie 7 for RhoC, GTPase activity was probed in reporter windows which followed the edge during protrusion and retraction events. For each window we obtained a time series of edge velocity and a time series of GTPase activity, allowing us to determine by Pearson’s cross-correlation the tightness of coupling and the time lag between protein activity and edge motion. Importantly, as the correlation is computed locally, these relationships are captured despite the asynchronous motion of different edge sectors. This analysis is completely invariant with respect to cell shape and largely insensitive to the heterogeneity of morphodynamic behaviors between cells. However, the correlation analysis from only a single window would be too noisy to determine unambiguous relationships. Therefore, we take all windows from all cells and perform a cubic spline fit to obtain the mean correlation, followed by 2000 bootstrap samplings of the residuals from per-window correlations relative to spline, to obtain the confidence intervals about the mean (RhoA: n=16 cells, 993 windows, RhoC: n=16 cells, 869 windows). This procedure was repeated for windows at different distances from the edge, allowing us to determine how the correlation changes with the location of the signaling activity. The second step in computational multiplexing is to compare the correlation functions of multiple Rho GTPases. As each of the functions uses the edge velocity as a reference, the differences between the functions indicate directly spatiotemporal differences between the Rho GTPases. 

Using this approach we first sought to identify subcellular regions where RhoA and RhoC differed during protrusion. In agreement with previous data [9,10], RhoA activity showed statistically significant, maximal positive correlation with edge velocity at a zone 0.9-1.9 μm from the cell edge ( *r* ~0.15), with a 29 sec (confidence interval, +/- 7 sec) mean time lag (Figure 3B, left). Analysis of RhoA activity in reporter windows successively further from the cell edge revealed decreasing yet still positive correlation (Figure 3B,C). The time lags steadily decreased to more negative values further from the edge (Figure 3B, D). This means that with greater distance from the edge RhoA activity is modulated with a delay relative to edge motion, consistent with the notion that active RhoA molecules or upstream activators (e.g. GEFs) diffuse from the site of initial activation at the cell edge.

RhoC also showed strongest correlation with edge velocity at 0.9-1.9 μm from the cell edge, with a time lag not statistically different from that observed for RhoA (r~0.125, Figure 3B, right). This positive correlation with edge velocity for RhoC mimics the trend observed for RhoA, decreasing with distances greater than 1.9 μm from the edge. Interestingly, while the time lag between RhoA activation and edge velocity remained negative regardless of spatial zone, RhoC by contrast had negative time lag values at the cell edge but positive time lag values at all zones measured greater than 0.9 μm from the cell edge (Figure 3B right, Figure 3D). This indicated that in this region RhoC activity is modulated slightly before a corresponding modulation in edge motion, while RhoA activity is modulated afterwards. Together these data show that RhoA and RhoC are differentially regulated in cell protrusions, with distinct kinetics in five spatial zones defined by their distance from the cell edge.

A multitude of RhoA/C isoform-specific functions and differential RhoA/C regulation have been described in the context of oncogenesis and metastasis. During EMT, RhoA activation was shown to be attenuated with a concomitant increase in RhoC expression [28]. In metastatic prostate cancer, RhoC interacts preferentially with the kinase PKN3 [29]. p38 gamma MAPK regulates breast cancer cell migration by controlling the ubiquitination of RhoC but not RhoA [30]. Also in the breast cancer paradigm, alpha 2 beta 1 integrins were found to be modulated specifically by RhoC in MDAMB231 cells [31]. RhoA was found to be primarily cytoplasmic while predominant membrane localization was observed for RhoC in pancreatic cancer cells, resulting in an opposing effect on cell migration and invasion [32]. Finally, microRNAs have been ascribed to the direct or indirect regulation of RhoC expression [33], with analogous examples elucidated for RhoA [34,35].

Despite such increasing evidence for RhoA/C isoform-specific functions, other studies have provided evidence for A/C isoform functional redundancy. Biallelic loss of RhoA in mouse fibroblasts resulted in no significant actin cytoskeleton abnormalities, suggesting that RhoC can functionally compensate [36]. It is thought that RhoA and RhoC are equally regulated by most Rho GEFs without isoform discrimination, including MyoGEF in breast cancer cells [37]. As one example of common effectors, PRK2 kinase is downstream of both RhoA and RhoC to regulate junctional integrity [38].

It is likely that the utilization of different RhoA/C functions by cells is contextual, where tumor microenvironment as well as cell autonomous factors contribute. Our work described here provides evidence for RhoA/C functional divergence, and importantly, does so at the level of GTPase activity, as opposed to analysis solely at the level of protein or mRNA levels. The differential kinetics of activation revealed by RhoA and RhoC FLARE.sc here may reflect a prominent role for RhoC in regulating myosin contractility at actomyosin contraction modules [39], and timing of associated actomyosin network disassembly [40]. Clearly, the use of RhoC FLARE.sc and other approaches to examine RhoA/C activity must be extended to cancer cell lines and ultimately to *in vivo* models on oncogenesis for a comprehensive understanding of differential isoform functions.

It is important to note that the RhoC biosensor, like the other FLARE biosensors [20], is designed with its C-terminal hypervariable region intact and unencumbered by an attached fluorescent protein. This maintains interaction with guanosine dissociation inhibitors (GDI) and regulation of reversible membrane translocation. Biosensors where the C terminus is modified have proven useful as indicators of upstream GEF activity, but do not necessarily reflect all negative regulatory inputs. Such biosensors can produce FRET that indicates localized GEF activity where endogenous GTPase may not in fact be active. The ability of the FLARE sensors to report negative regulation can render cells sensitive to high levels of expression. Careful titration to determine appropriate levels is important [10,19]. Perhaps the most challenging aspect of biosensor imaging is maintaining cell health in the face of irradiation, manipulation and expression of ectopic protein. Dual chain biosensors [10] are in general more sensitive than single chain biosensors, because the single chain sensors’ FRET is not fully abrogated in the off state. In comparison to the dynamic range of the single chain RhoA FLARE (55.5% between WT vs. WT+GDI) [9] and the single chain RhoC FLARE biosensor described here (50.1% between WT vs WT+GDI; Figure 1B), dual chain biosensors have a very large range (ranging from no FRET to some positive value). The measurable range of dual chain biosensors is limited by the signal/noise of the imaging system. Improved dual chain designs are currently under development in our laboratories.

 In summary, we report here the development of RhoC FLARE.sc, a new single-chain biosensor for RhoC, capable of detecting the isoform-specific activation dynamics of Rho GTPases at unprecedented resolution during cell protrusion. Spatially, both RhoA and RhoC are maximally activated 0.9-1.9 µm from the cell edge during protrusion. These activations occur with different kinetics, in that RhoC activation occurs before protrusive events are initiated, while RhoA activation slightly lags behind the motion of the leading edge. These differences in activation dynamics begin to point to functionally divergent roles of these two close isoforms of Rho GTPases. Additional work will be necessary to identify the mechanisms by which the differential activation kinetics of RhoA and RhoC are orchestrated during cell motility. The identification of additional GEFs that discriminate between RhoA and RhoC, as well as the identification of RhoC-specific effectors like FMLN2 [41] and MLK-related kinase [42], will aid in elucidating these mechanisms.

## Supporting Information

Appendix S1 **Biosensor base sequence and corresponding amino acid sequence.**
(DOC)

Figure S1 **Biosensor domain diagram and amino acid sequence.**
(TIF)

Movie S1 **RhoA activity at the edge of a MEF cell plated on 5 µg/ml fibronectin.** Activity is maximal at the region immediately adjacent to the cell edge and largely restricted to this region. Duration of original sequence: 30 min. Magnification 40X, 2X2 binning. Frame interval: 60 sec. Replay: 10 frames/sec. Scale bar: 10 µm. Color bar defines the dynamic range of the FRET/CFP ratio. See Figure 2A.
(MOV)

Movie S2 **Additional example of RhoA activity at the edge of a MEF cell plated on 5 µg/ml fibronectin.** Duration of original sequence: 30 min. Magnification 40X, 2X2 binning. Frame interval: 60 sec. Replay: 5 frames/sec. Scale bar: 10 µm. Color bar defines the dynamic range of the FRET/CFP ratio. See Figure 2A.
(MOV)

Movie S3 **RhoC activity at the edge of a MEF plated on 5 µg/ml fibronectin.** Activation is seen both at the edge and at variable regions within the cell. Duration of original sequence: 20 min. Magnification 40X, 2X2 binning. Frame interval: 10 sec. Replay: 10 frames/sec. Scale bar: 10 µm. Color bar defines the dynamic range of the FRET/CFP ratio. See Figure 2A.
(MOV)

Movie S4 **Additional example of RhoC activity at the edge of a MEF plated on 5 µg/ml fibronectin.** Duration of original sequence: 20 min. Magnification 40X, 2X2 binning. Frame interval: 10 sec. Replay: 10 frames/sec. Scale bar: 10 µm. Color bar defines the dynamic range of the FRET/CFP ratio. See Figure 2A.
(MOV)

Movie S5 **RhoA activity and corresponding DIC of a MEF undergoing macropinocytosis.** Note burst of activation accompanying vesicle closure. Duration of original sequence: 30 min. Magnification 40X, 2X2 binning. Frame interval: 60 sec. Replay: 5 frames/sec. Scale bar: 10 µm. Color bar defines the dynamic range of the FRET/CFP ratio. See Figure 2B.
(MOV)

Movie S6 **RhoC activity and corresponding DIC of a MEF undergoing macropinocytosis.** Note activation within actin structures preceding closure. Duration of original sequence: 45 min. Displaying frames #145-245. Magnification 40X, 2X2 binning. Frame interval: 10 sec. Replay: 10 frames/sec. Scale bar: 10 µm. Color bar defines the dynamic range of the FRET/CFP ratio. See Figure 2B.
(MOV)

Movie S7 **Overlay of sampling windows and velocity vectors for a MEF expressing the RhoC biosensor.** For correlation of RhoC activity and cell edge velocity, 2.5 µm width, 0.9 µm depth sampling windows (yellow) are placed at fixed distances relative to the edge. Green lines indicate velocity vectors. Duration of original sequence: 20 min. Magnification 40X, 2X2 binning. Frame interval: 10 sec. Replay: 10 frames/sec. See Figure 3A.
(MOV)
