# Supplementary material for: A RhoC Biosensor Reveals Differences in the Activation Kinetics of RhoA and RhoC in Migrating Cells
Source: PLoS One. 2013 Nov 5;8(11):e79877. doi: 10.1371/journal.pone.0079877 (PMC3818223; doi:10.1371/journal.pone.0079877)
Supplement: Appendix S1 — Biosensor base sequence and corresponding amino acid sequence. (DOC) [file pone.0079877.s001.doc]

**Appendix 1: RhoC FLARE.SC**

**N terminus**

**ROCK RBD**

atggcgcgaggccttctggaagaacagtattttgaattgacgcaagaaagcaagaaagctgcttcaagaaatagacaagagattacagataaagatcacactgttagtcggcttgaagaagcaaacagcatgctaaccaaagatattgaaatattaagaagagagaatgaagagctaacagagaaaatgaagaaggcagaggaagaatataaactggagaaggaggaggagatcagtaatcttaaggctgcctttgaaaagaatatcaacactgaacgaacccttaaaacacaggctgttaacaaattggcagaaataatgaatcgaaaagattttaaaattgatagaaagaaagctaatacacaagatttgagaaagaaagaaaaggaaaatcgaaagctgcaactgGaactcaaccaagaaagagag

**linker**

ggatccgga

**monomeric Cerulean**

atggtgagcaagggcgaggagctgttcaccggggtggtgcccatcctggtcgagctggacggcgacgtaaacggccacaagttcagcgtgtccggcgagggcgagggcgatgccacctacggcaagctgaccctgaagttcatctgcaccaccggcaagctgcccgtgccctggcccaccctcgtgaccaccctgacctggggcgtgcagtgcttcgcccgctaccccgaccacatgaagcagcacgacttcttcaagtccgccatgcccgaaggctacgtccaggagcgcaccatcttcttcaaggacgacggcaactacaagacccgcgccgaggtgaagttcgagggcgacaccctggtgaaccgcatcgagctgaagggcatcgacttcaaggaggacggcaacatcctggggcacaagctggagtacaacgccatcagcgacaacgtctatatcaccgccgacaagcagaagaacggcatcaaggccaacttcaagatccgccacaacatcgaggacggcagcgtgcagctcgccgaccactaccagcagaacacccccatcggcgacggccccgtgctgctgcccgacaaccactacctgagcacccagtccaagctgagcaaagaccccaacgagaagcgcgatcacatggtcctgctggagttcgtgaccgccgccgggatcactctcggcatggacgagctgtacaaa

**LINKER**

agcttaacttctggttctggtaaacctggttctggtgaaggttctactaaaggtggatctacttctggttctggtaaacctggttctggtgaaggttctactaaaggtggatctacttctggttctggtaaacctggttctggtgaaggttctactaaaggtggatctacttctggttctggtaaacctggttctggtgaaggttctactaaaggtggatctgcggccgca

**MONOMERIC VENUS** atggtgagcaagggcgaggagctgttcaccggggtggtgcccatcctggtcgagctggacggcgacgtaaacggccacaagttcagcgtgtccggcgagggcgagggcgatgccacctacggcaagctgaccctgaagttgatctgcaccaccggcaagctgcccgtgccctggcccaccctcgtgaccaccctcggctacggcctgcagtgcttcgcccgctaccccgaccacatgaagcagcacgacttcttcaagtccgccatgcccgaaggctacgtccaggagcgcaccatcttcttcaaggacgacggcaactacaagacccgcgccgaggtgaagttcgagggcgacaccctggtgaaccgcatcgagctgaagggcatcgacttcaaggaggacggcaacatcctggggcacaagctggagtacaactacaacagccacaacgtctatatcatggccgacaagcagaagaacggcatcaaggcaaacttcaagatccgccacaacatcgaggacggcggcgtgcagctcgccgaccactaccagcagaacacccccatcggcgacggccccgtgctgctgcccgacaaccactacctgagctaccagtccaaactgagcaaagaccccaacgagaagcgcgatcacatggtcctgctggagttcgtgaccgccgccgggatcactctcggcatggacgagctgtacaag

**LINKER**

gaattcaca

**RHOC**

atggctgccatccggaagaaactggtgatcgttggggatggtgcctgtgggaagacctgcctcctcatcgtcttcagcaaggatcagtttccggaggtctacgtccctactgtctttgagaactatattgcggacattgaggtggacggcaagcaggtggagctggctctgtgggacacagcagggcaggaagactatgatcgactgcggcctctctcctacccggacactgatgtcatcctcatgtgcttctccatcgacagccctgacagcctggaaaacattcctgagaagtggaccccagaggtgaagcacttctgccccaacgtgcccatcatcctggtggggaataagaaggacctgaggcaagacgagcacaccaggagagagctggccaagatgaagcaggagcccgttcggtctgaggaaggccgggacatggcgaaccggatcagtgcctttggctaccttgagtgctcagccaagaccaaggagggagtgcgggaggtgtttgagatggccactcgggctggcctccaggtccgcaagaacaagcgtcggaggggctgtcccattctc

**c terminus**
