# Supplementary figures and images for: A RhoC Biosensor Reveals Differences in the Activation Kinetics of RhoA and RhoC in Migrating Cells
Source: PLoS One. 2013 Nov 5;8(11):e79877. doi: 10.1371/journal.pone.0079877 (PMC3818223; doi:10.1371/journal.pone.0079877)

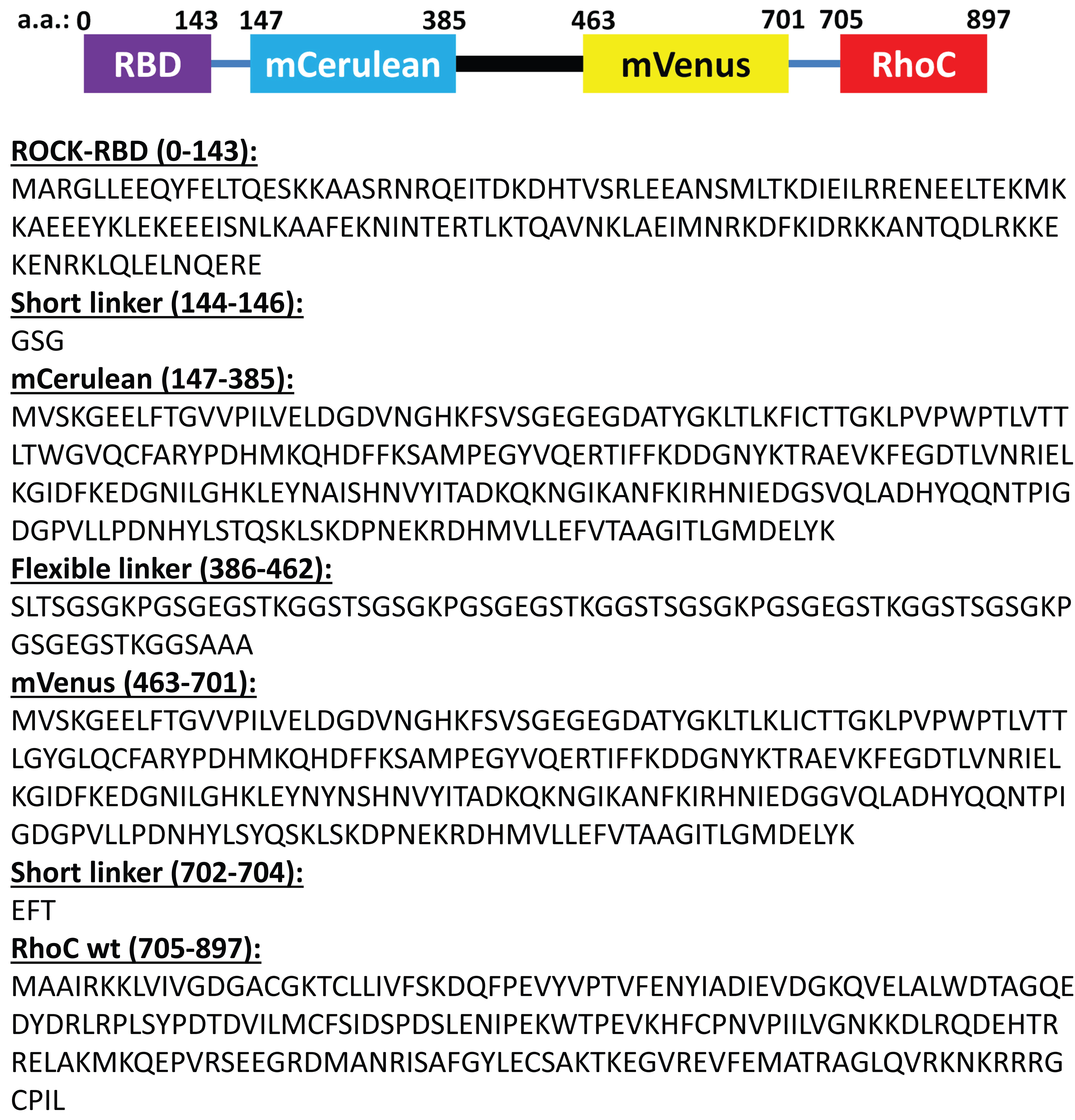

Supplement: Figure S1 — Biosensor domain diagram and amino acid sequence. (TIF) [file pone.0079877.s002.tif]
